# Supplementary material for: An integrated Mg battery-powered iontophoresis patch for efficient and controllable transdermal drug delivery
Source: Nat Commun. 2023 Jan 18;14:297. doi: 10.1038/s41467-023-35990-7 (PMC9849227; doi:10.1038/s41467-023-35990-7)
Supplement: Supplementary file 1 — Supplementary Information [file 41467_2023_35990_MOESM1_ESM.pdf]

Supplementary Information

An integrated Mg battery-powered iontophoresis patch for efficient and controllable transdermal drug delivery

Yan Zhou[1], Xiaoteng Jia[2*], Daxin Pang[3], Shan Jiang[1], Meihua Zhu[1], Geyu Lu[2,4], Yaping Tian[5*], Caiyun Wang[6*], Danming Chao[1*], Gordon Wallace[6]

[1]College of Chemistry, Jilin University, Changchun 130012, China.

[2]State Key Laboratory of Integrated Optoelectronics, College of Electronic Science and Engineering, Jilin University, Changchun 130012, China.

[3]College of Animal Sciences, Jilin University, Changchun 130062, China.

[4]International Center of Future Science, Jilin University, Changchun 130012, China.

[5]Department of Dermatology and Venerology, The First Hospital of Jilin University, Changchun 130021, China.

[6]ARC Centre of Excellence for Electromaterials Science, Intelligent Polymer Research Institute, AIIM Facility, University of Wollongong, North Wollongong, NSW 2500, Australia.

Supplementary Table 1 Summary of current iontophoresis systems.

Category	Typical materials	Output	Model drug	Release capacity ($\mu g\ cm^{-2}$)	Application	Ref.
Direct current (DC)	—	0.3 mA	Dex	46.15	Study the competition of chloride released from Ag/AgCl cathode on the iontophoretic delivery.	1
	Microneedle array	0.5, 1.0 mA	Insulin nanovesicle	—	A transdermal patch can achieve synergistic and remarkable enhancement of drug delivery with precise electronic control.	2
	Poly(ethylene glycol) hydrogel	100 mA cm^{-2}	Dextran/ Bevacizumab	1443 / 900	An iontophoresis device demonstrates high-efficiency intraocular delivery.	3
	PPy nanoparticles	0.13 mA cm^{-2}	Insulin	68.29	Investigate PPy nanoparticles for controlled transdermal iontophoresis of insulin.	4
PENG	Polylactic acid-gold-PPy microneedles	100 V, 2 μA	Dex	4.3	Collect and convert biomechanical energy into electrical energy to control drug release for psoriasis treatment.	5
TENG	DOX loaded red blood cells on the Cu film	70 V, 0.5 μA	DOX	—	Killing cancer cells in vitro and in vivo at a low drug dosage.	6
	Poly(3-hexylthiophene) films	647 V, 165 μA	Salicylic acid	—	TENG can provide a steady voltage supply for sustainable drug release.	7
	PPy/Dex film on gold electrode	100 V, 19 mW cm^{-2}	Dex	35	Electricity generated from TENG was used to power iontophoresis treatment.	8
	Silicon nanoneedles-array	20 V, 4 μA	siRNA/ dextran-FITC	688	TENG-driven electroporation system is developed for intracellular drug delivery in vivo and in vitro.	9
	Poloxamer hydrogel	1200 V, 20 μA	Rhodamine 6G/ methylene blue	2.63 /11.3	A wearable TENG is used as the motion sensor and energy harvester for iontophoresis.	10
	Polydimethylsiloxane drug reservoir	15 V, 1.5 mA	Fluorescent particles	—	TENG-based self-powered implantable drug-delivery system demonstrates its functionality for ocular drug delivery.	11

Biofuel cell	Biocathode: PEDOT functionalized gold electrode<br>Bioanode: Carbon nanotubes	0.4 V,<br>33 mW cm^{-2}	Acetaminophen	—	A biocomputing, logic-based detection method with a controlled-release drug delivery actuator.	12
	Biocathode: PEDOT doped NPG/Os(bpy)$_2$PVI-BOx<br>Bioanode: NPG/Os-(bpy)$_2$PVI-GOx	0.377 V,<br>1.35 µW cm^{-2}	Ibuprofen/ Fluorescein/ diamidino-phenylindole	197 /0.101/ 0.102	A self-powered, controlled drug-release system based on bilayer-modified electrodes has been demonstrated.	13
	Biocathode: BOD-carbon fabrics<br>Bioanode: FDH-carbon fabrics	0.55-0.7 V,<br>10-50 µA cm^{-2}	Rhodamine B<br>Ascorbyl glucoside	—	BFC-driven current-assisted penetration of ascorbyl glucoside and rhodamine B into the skin.	14
	Biocathode: PSp/carbon sphere /glassy carbon<br>Bioanode: PDS/gold nanobowl/ glassy carbon	145 µW cm^{-2}	DOX	1.15	A robust glucose/O_2 fuel cell-based biosensor is integrated with a targeted drug delivery system to create a self-sustained and highly compact drug delivery model.	15
	Biocathode: BOD-immobilized electrode<br>Bioanode: FADGDH immobilized electrode	0.40 V<br>157 µW cm^{-2}	—	—	An autonomous, self-powered, sensing actuator that employs the principle of bio-capacitor as the core technology.	16
Battery	Cathode: PEDOT<br>Anode: Zn	—	Rhodamine B	—	A battery-driven drug delivery device powered by physiological pH can target intended sites and be actuated galvanically to trigger localized drug release.	17
	Cathode: PPy<br>Anode: Mg	—	Adenosine triphosphate (ATP)	—	The drug release from this system can be performed without the external power source, and the massive drug release only occurs at around human body temperature.	18
	Cathode: AgCl<br>Anode: Zn<br>Drug-loading material: PEDOT /PAAm hydrogel	1.08 V, 0.2 mA,<br>60 µW	Rhodamine B	—	Iontophoretic drug delivery device, and an electrical nerve stimulator, for transcutaneous bioanalysis and therapy.	19

	Cathode: PEDOT<br>Anode: Zn	—	Gold nanoparticle	—	In vivo studies of PEDOT/Zn artificial micromotors demonstrate their distribution, retention, and toxicity.	20
	Cathode: P(AM-co-SV) hydrogel<br>Anode: Mg	1.1 V, 10 mA cm^{-2}, 3.57 mWh cm^{-2}	Dex	139.6	Battery-powered iontophoresis generated an on-demand transdermal release profile without complicated feedback circuits.	**This work**

Notes:

DC: direct current; PENG: piezoelectric nanogenerator; TENG: triboelectric nanogenerator; BFC: enzymatic biofuel cell;

NPG/Os(bpy)$_2$PVI-BOx: Os(bpy)$_2$PVI-mediated bilirubin oxidase (BOx); NPG/Os-(bpy)$_2$PVI-GOx: [Os(2,2'-bipyridine)$_2$(polyvinylimidazole)$_{10}$Cl]$^{+/2+}$ (Os(bpy)$_2$PVI)-mediated glucose oxidase (GOx);

BOD: bilirubin oxidase; FDH: fructose dehydrogenese; PSp: phosphatidylserinebinding peptide; PDS: partial complementary DNA double strand-decorated; FADGDH: flavin adenine dinucleotide-dependent glucose dehydrogenase;

PPy: polypyrrole; PEDOT: poly(3,4-ethylenedioxythiophene); PANI: polyaniline; PAAm: polyacrylamide;

Dex: Dexamethasone sodium phosphate; DOX: Doxorubicin; —: not provided.

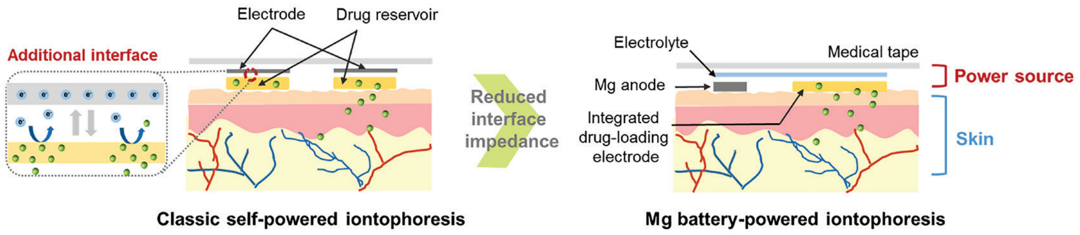

Supplementary Fig. 1 Comparison of classic self-powered iontophoresis and Mg battery-powered iontophoresis in this work. System complexity is decreased using viologen-based hydrogels as an integrated drug-loading electrode, thus avoiding additional interface impedance in the conventional design of separate electrodes and drug reservoirs.

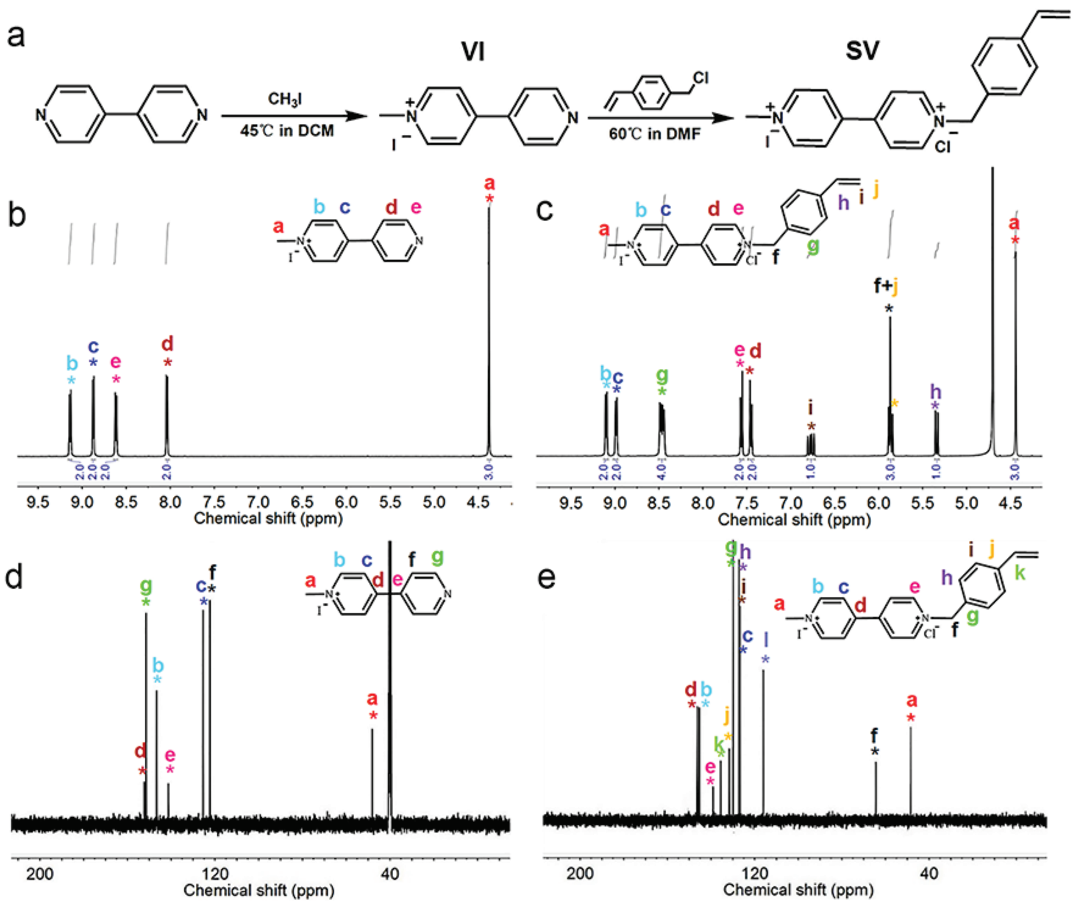

Supplementary Fig. 2 Synthetic routes and NMR of viologen monomers. **a** Synthesis of viologen monomers. **b** ^{1}H NMR and **d** ^{13}C NMR of VI monomer in DMSO-d_6. **c** ^{1}H NMR and **e** ^{13}C NMR of SV monomer in D_2O. "⋆" represents the NMR peak position maker.

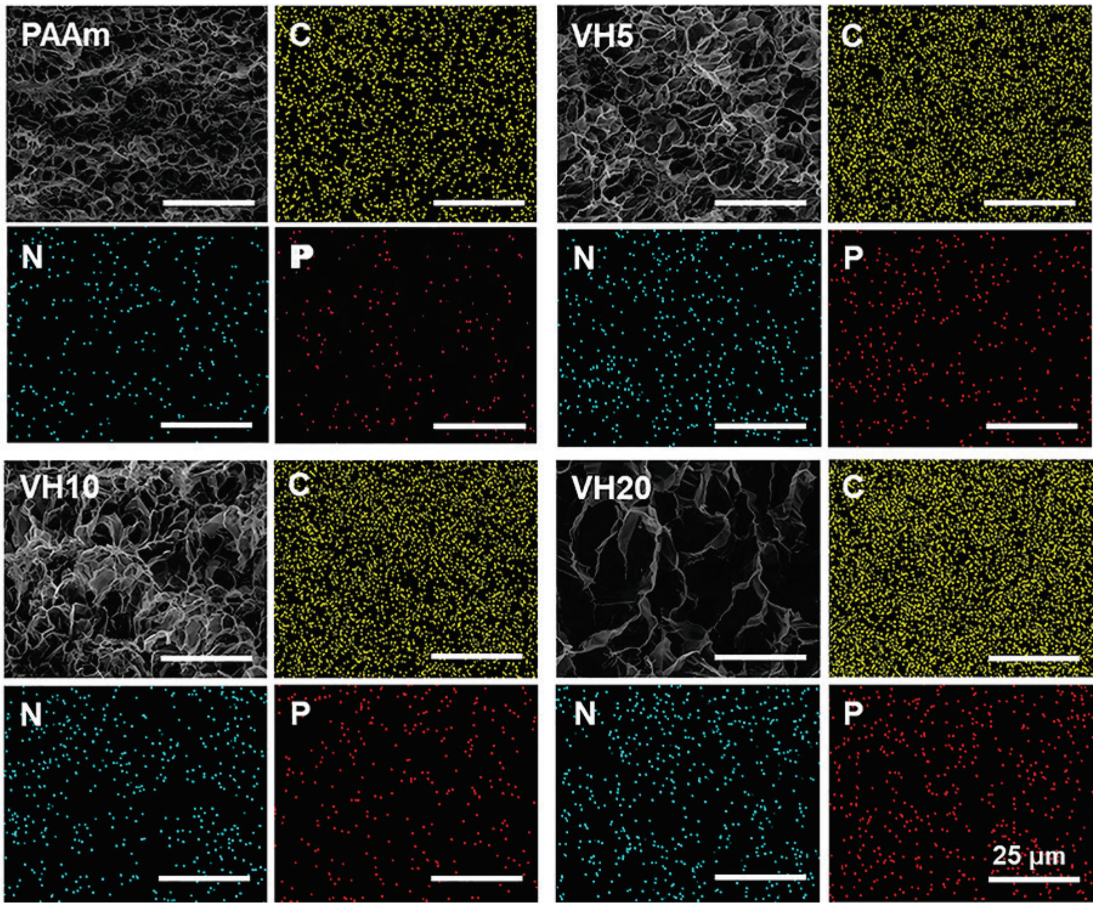

Supplementary Fig. 3 SEM and corresponding EDS images of the Dex-loaded P(AM-co-SV) hydrogels. Measurements were repeated three times independently with similar results.

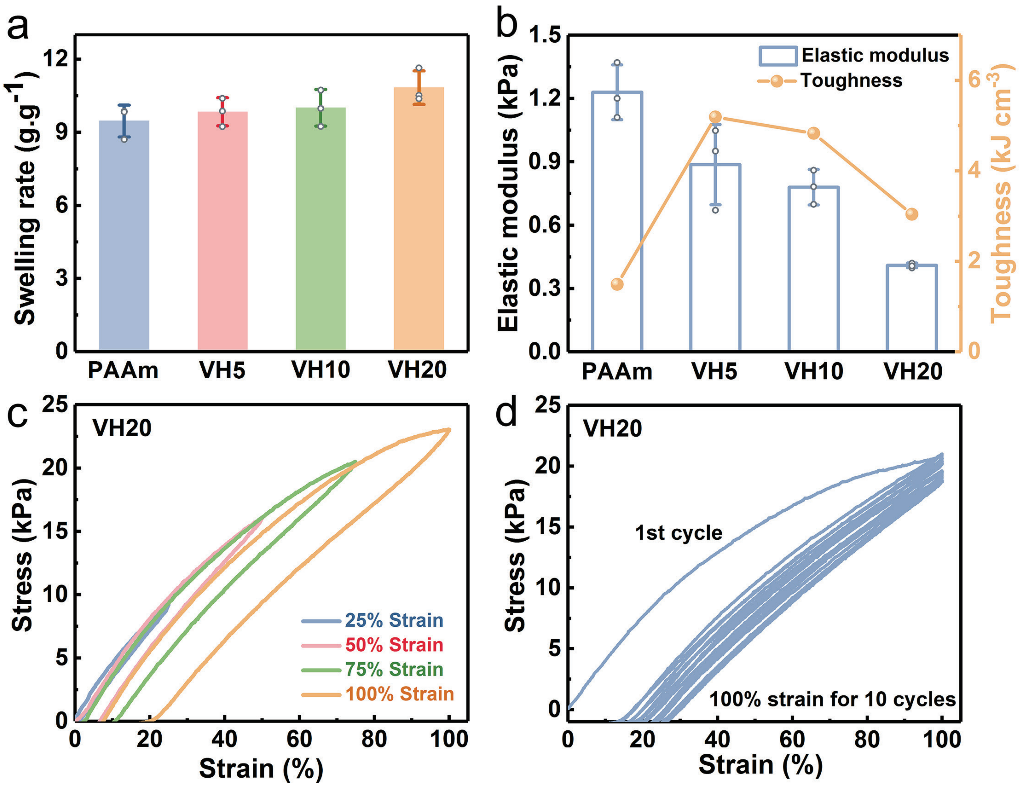

Supplementary Fig. 4 The swelling rate of P(AM-co-SV) hydrogels and cyclic tensile curves of VH20. **a** Maximum swelling rate of P(AM-co-SV) hydrogels in 25 °C PBS solution (n = 3 independent experiments). **b** Elastic modulus and toughness of P(AM-co-SV) hydrogels (n = 3). **c** Cyclic tensile curves of VH20 hydrogel with increasing tensile. **d** 10-cyclic tensile curves of VH20 hydrogel. Data are presented as mean ± SD in **(a, b)**.

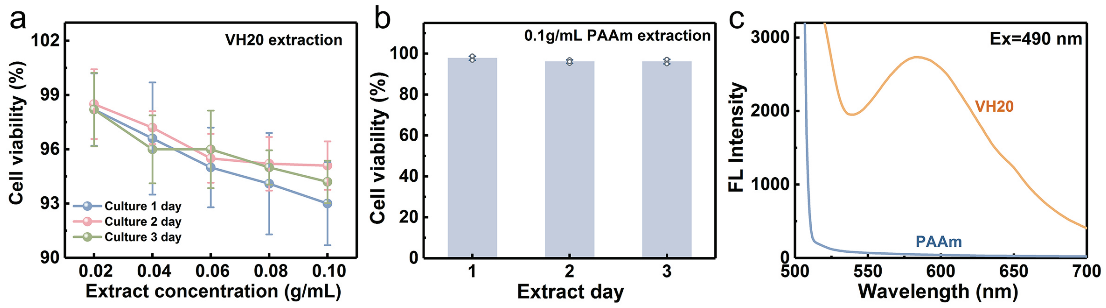

Supplementary Fig. 5 The biocompatibility of VH20 hydrogel. **a** Cell viability of L929 cells cultured with different concentrations of VH20 hydrogel extracts for 1, 2, and 3 days (n = 3 independent experiments). **b** Cell viability of L929 cells cultured with PAAm hydrogel extracts (0.1 g/mL) for 1, 2, and 3 days (n = 3 independent experiments). **c** Fluorescence emission spectra of PAAm and VH20 hydrogels at an excitation wavelength of 490 nm. Data are presented as mean ± SD in **(a, b)**.

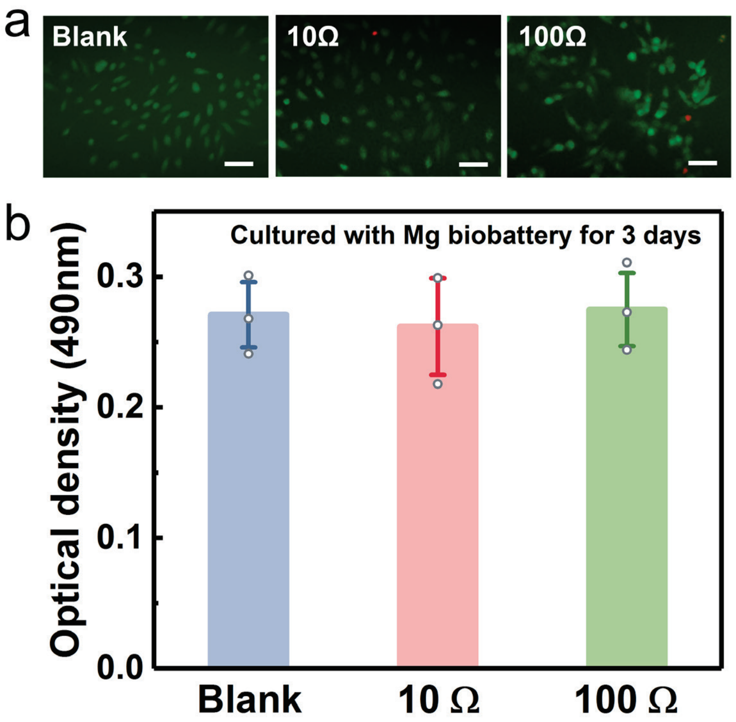

Supplementary Fig. 6 The biocompatibility of Mg battery. **a** Fluorescence graphs and **b** Optical density of L929 cells cultured on VH20 hydrogel side of Mg battery after 3 days, accompanied by discharge for 30 min/per day when loaded with 10 Ω and 100 Ω resistors, respectively (n = 3 independent experiments). Scale bar: 100 µm. Data are presented as mean ± SD.

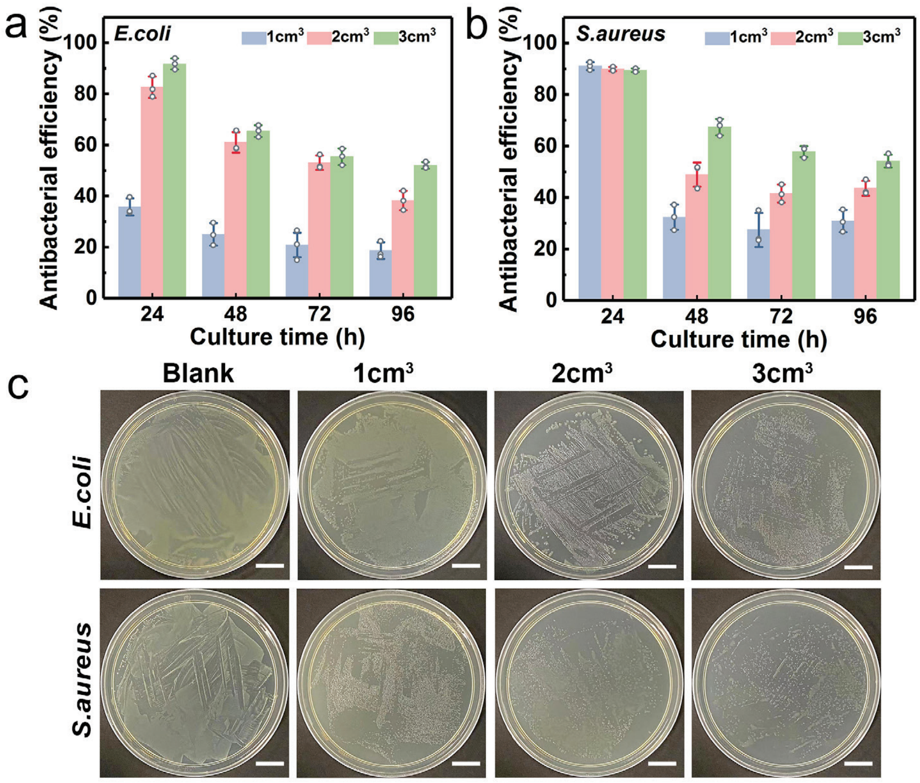

Supplementary Fig. 7 Antibacterial efficiency of VH20 hydrogels co-cultured with **a** *E. coli*. and **b** *S. aureus* (n=3). **c** Plate photographs of *E. coli* and *S. aureus* co-cultured with different volumes of VH20 hydrogels. Scale bar: 1 cm. Data are presented as mean ± SD in **(a, b)**.

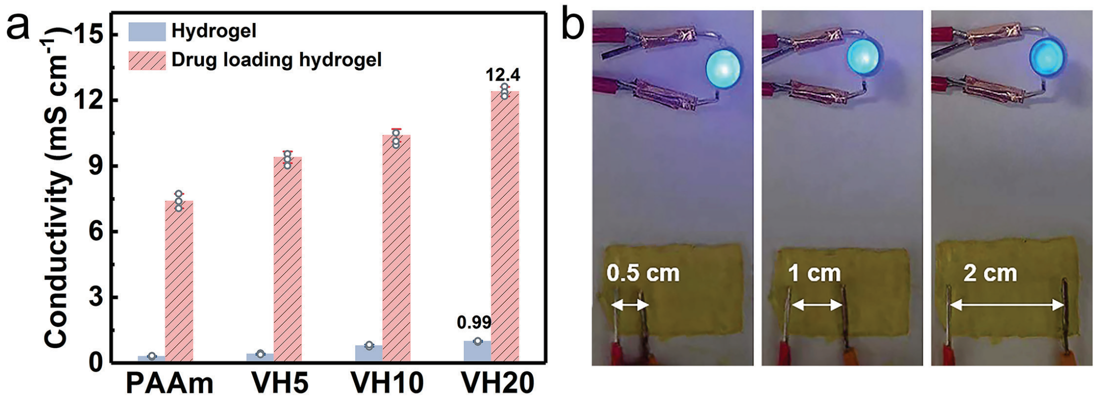

Supplementary Fig. 8 Ionic conductivity properties of P(AM-co-SV) hydrogels. **a** Ionic conductivity of P(AM-co-SV) hydrogels with and without Dex loading. The length, width, and height of the hydrogel are 6 cm, 2 cm, and 0.3 cm, respectively (n = 3 independent experiments). Data are presented as mean ± SD. **b** Photographs of LED bulbs (3 mm, 2.2-2.4V) connected with VH20 hydrogels at distances of 0.5 cm, 1 cm, and 2 cm, respectively.

Supplementary Table 2 Composition and properties of P(AM-co-SV) hydrogels.

Sample	SV (g)	AM (g)	Drug-loading capacity (mg/g)	Swelling ratio (g/g)	Elastic modulus (kPa)	Toughness (KJ/cm^3)	Ionic conductivity (mS/cm)	Tissue impedance (Ω, @1Hz)
**PAAm**	0	1.8	1.81	9.46	1.23	1.50	0.30	3.2×10^6
**VH5**	0.095	1.8	2.51	9.84	0.89	5.19	0.40	—
**VH10**	0.20	1.8	3.92	10.01	0.78	4.83	0.79	—
**VH20**	0.45	1.8	7.16	10.83	0.41	3.04	0.99	4.3×10^5

Notes

—: not provided.

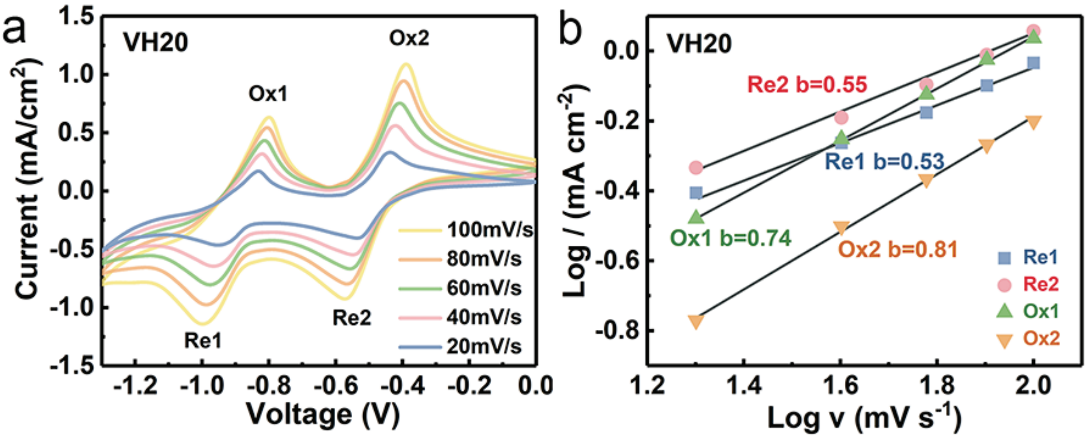

Supplementary Fig. 9 Electrochemical properties of VH20 hydrogel. **a** CV curves of VH20 hydrogel at scan rates of 20, 40, 60, 80, and 100 mV/s, respectively. **b** Fitting curves and b values of VH20 hydrogel.

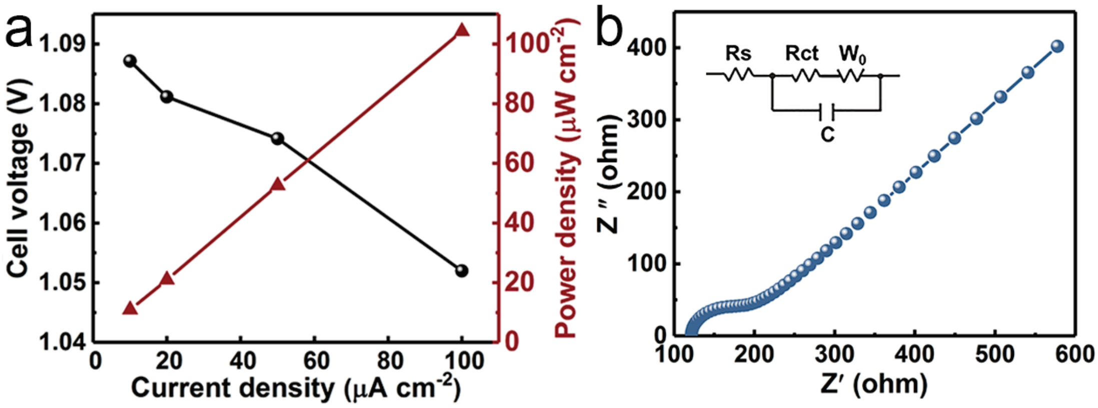

Supplementary Fig. 10 Electrochemical properties of Mg biobattery. **a** Cell voltage from the middle point of discharge curve and **b** EIS of Mg biobatteries with VH20 hydrogel cathode.

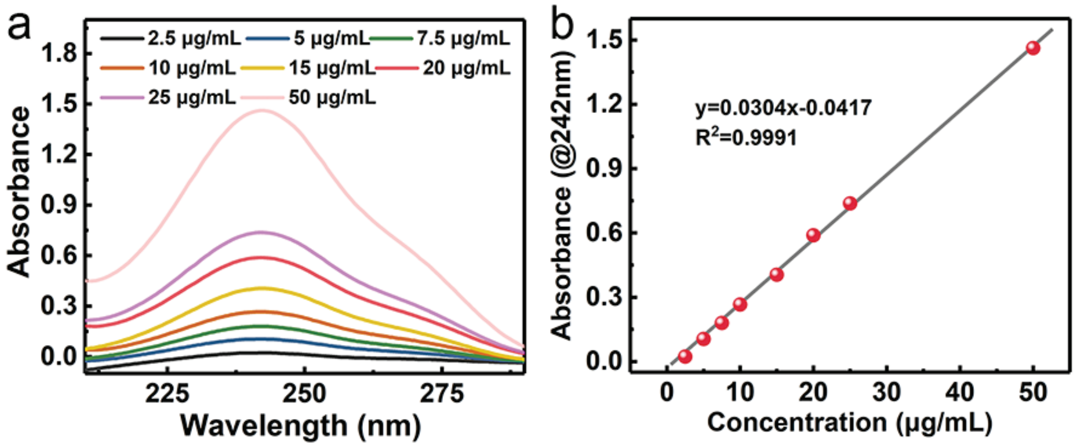

Supplementary Fig. 11 Establishment of the standard curve for Dex solution. **a** UV-Vis absorption spectra of different concentrations of Dex in PBS solution. **b** Standard curve of Dex in PBS solution at 242 nm.

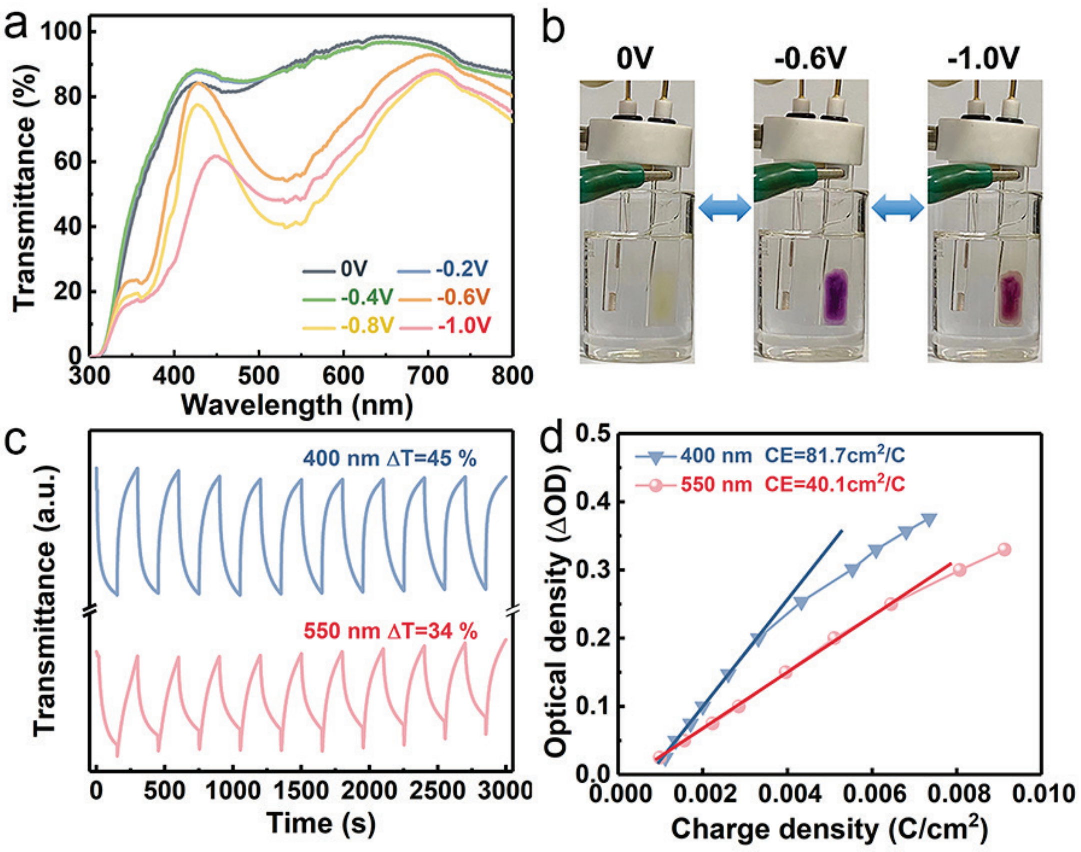

Supplementary Fig. 12 Electrochromic properties of VH20 hydrogel. **a** Transmittance spectrum of VH20 electrode in 0.01 M PBS solution at different potentials (-1.0-0 V). **b** Photographs of VH20 electrode at different potentials. **c** Transmittance changes of VH20 electrode monitored at 400 nm and 550 nm. The residence time was fixed at 300 s. **d** Coloration efficiency of the VH20 electrode at 400 nm and 550 nm.

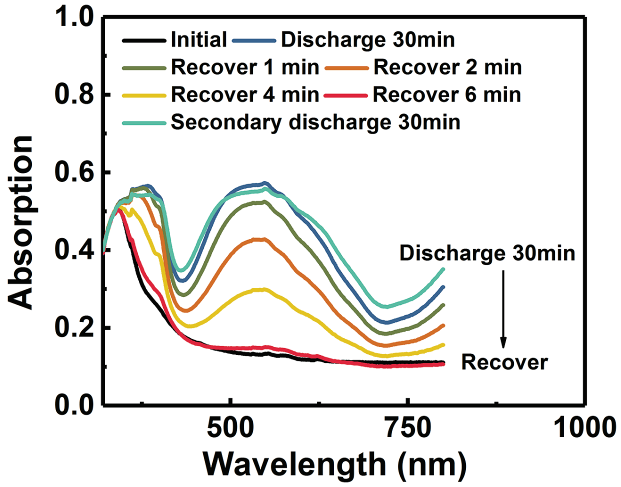

Supplementary Fig. 13 Absorption spectra of the VH20 electrode at different recovery times after the Mg battery was discharged for 30 min.

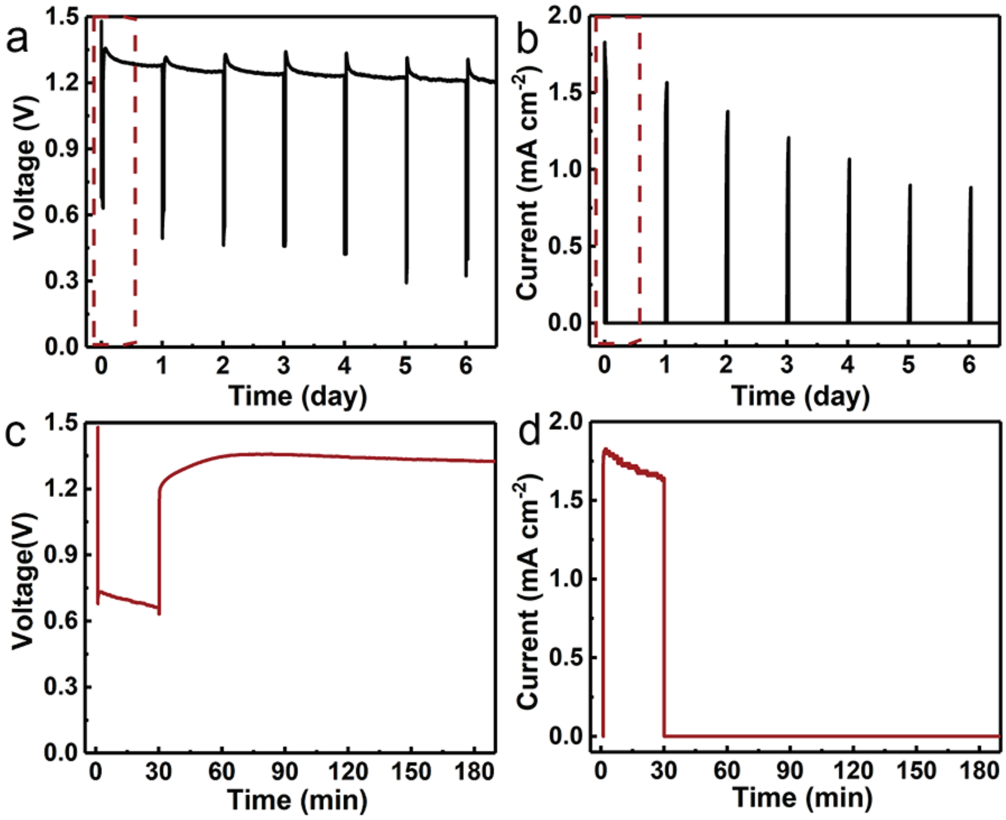

Supplementary Fig. 14 Current and voltage monitoring of iontophoresis patch. **a** Voltage and **b** current profiles during the controlled intermittent release of Dex from iontophoresis patch over 6 days. **c** Enlarged voltage and **d** current profiles during the first 3 hours.

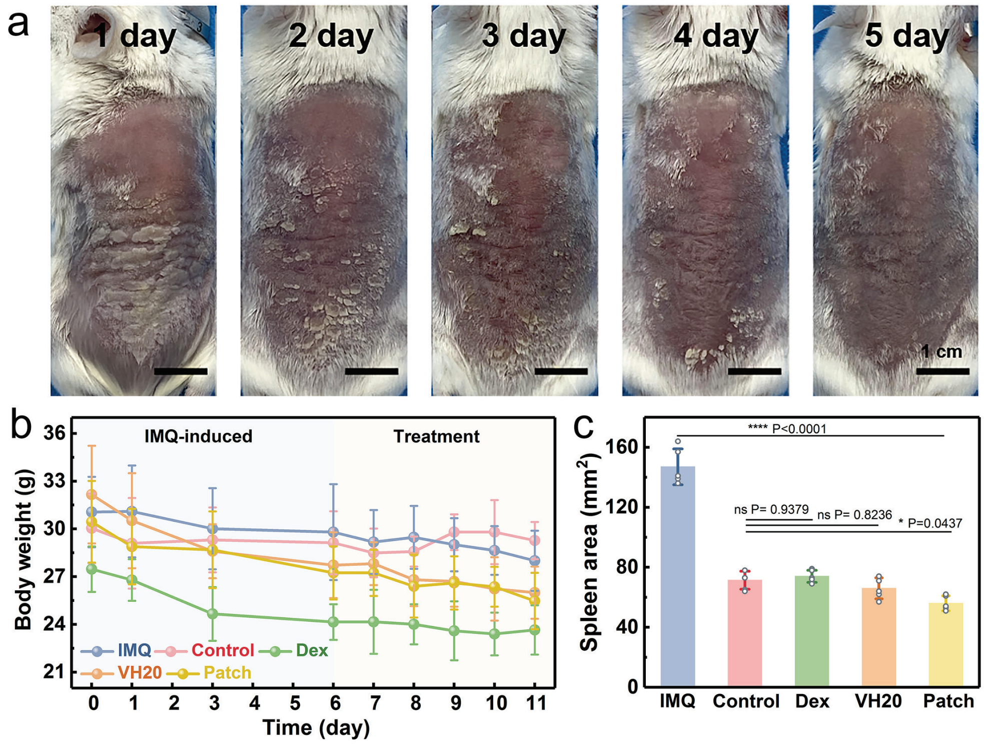

Supplementary Fig. 15 Therapeutic process of iontophoresis patch on mouse psoriasis model. **a** Photographs of the mice's skin during treatment with an iontophoresis patch (with 30 min of electrical stimulation every day). **b** Body weight of mice during IMQ-induced and different treatments (n = 5 biologically independent animals). **c** Spleen area of mice at the end of different treatments (n = 5 biologically independent animals). Data are presented as mean ± SD in **(b, c)**. * $P<0.05$, ** $P<0.01$, *** $P<0.001$, **** $P<0.0001$, ns for no significance, P value was generated by one-way analysis of variance (ANOVA), followed by Tukey's multiple-comparison post hoc test.

Supplementary Table 3 Properties and applications of conductive hydrogels for electrically stimulated drug delivery

Sample	Driven voltage	Model drug/ Loading capacity	Swell ratio (g/g)	Elastic modulus	Conductivity (mS/cm)	Tissue impedance (Ω, @1Hz)	Biological experiment	Application	Ref.
PPy-Poly(vinyl alcohol)	1.9V	Rosiglitazone/ 3.3 mg	6	120 kPa	0.08	—	Cell culture Animal	Transdermal delivery is driven by reverse electrodialysis battery.	21
PPy-PAAM/CS	-3V and -1V	Dex/ 2 mg g^{-1}	3	7.5-50.1 MPa	3	—	Cell culture Animal	Hydrogel accelerates wound healing.	22
PEDOT:PSS -PAAm	1.08V	Rhodamine B/ —	—	8 kPa	—	~5×10^5	Animal	Tissue-like iontophoretic device.	19
PEDOT-PDMAAp	CV -0.5V-0.5V	Dex/ —	—	—	—	—	—	Promote efficient release of Dex and fluorescein.	23
PEDOT-PEG/PLGA	—	Cyclosporin A/ 458.4 μg	—	—	—	—	Cell culture Animal	Implanted neural electrode surface.	24

Aniline-CS/Agarose	—	Dex/ 50 mg	8	—	0.1	—	Cell culture	Dex is released under the DC electrical stimulation.	25
Aniline-Hexamethylene diisocyanate	3V and 1V	Dex/ 1.5 mg mL^{-1}	0.33	—	0.08	—	Cell culture Animal	Dex is released under the DC electrical stimulation.	26
PANI-CS/ Polyethylene glycol	3V	Deferoxamine/ 2.42 mg mL^{-1}	>20	1.7 kPa	0.26	—	Antibacterial Cell culture Animal	On treating infected chronic wounds.	27
PANI-CS	3V and 1V	Ibuprofen/ 1.5 mg mL^{-1}	10-14	—	0.69	—	Antibacterial Cell culture Animal	Electrical field and pH responsiveness drug delivery system.	28
Graphene-Polymethacrylic acid	10V	DOX/ —	30	—	—	—	Cell culture Animal	Controlled drug release at low voltage in a pulsed manner.	29
CNT-Gelatin	9V	Diclofenac/ 19.8 mg	7.5	—	—	—	Cell culture	Electro-stimulated release.	30
CNT-CS /pNIBBIm	—	Ketoprofen/ 3.86 mg mg^{-1}	0.352	—	1.27	—	Cell culture	Electrical and thermal switches on/off.	31

Tetraethoxysilane-CS	15V-60V	Ethosuximide/ 15 mg mL^{-1}	4.27	—	—	—	—	Electro-regulated drug release profile.	32
VH20	-1V and -0.6V	Dex/ 7.16 mg g^{-1}	10.83	0.41 kPa	0.99	4.3×10^{5}	Antibacterial<br>Cell culture<br>Animal	Iontophoresis patch for transdermal drug delivery.	**This work**

Notes

PPy: polypyrrole; PEDOT: poly(3,4-ethylenedioxythiophene); PSS: polystyrene sulfonate; PANI: polyaniline; CNT: carbon nanotube

PAAm: polyacrylamide; CS: chitosan; PLGA: poly(lactic-co-glycolic) acid; PDMAAp: poly(dimethylacrylamide*co*-4-methacryloyloxy benzophenone-*co*-4-styrenesulfonate); pNIBBIm: poly(N-Isopropylacrylamide-co-3,3′-(Butane-1,4-diyl)bis(1-vinylimidazol-3-ium) bromide)

Dex: Dexamethasone sodium phosphate; DOX: Doxorubicin; —: not provided.

Supplementary Table 4 Drug release profiles of VH20 hydrogels in PBS driven by an external source and Mg battery.

	**Passive delivery**	**-1.0 V**	**-0.6 V**	**10^1 Ω**	**10^2 Ω**	**10^3 Ω**	**10^4 Ω**
**Continuous release for 3h (mg g^{-1})**	0.50	3.08	2.08	2.92	2.69	1.85	0.91
**Continuous release efficiency for 3h**	6.9%	43.0%	29.0%	40.7%	37.5%	25.8%	12.7%
**Intermittent release for 6d (mg g^{-1})**	—	—	—	6.38	5.10	3.36	—
**Intermittent release efficiency for 6d**	—	—	—	89.1%	71.2%	46.9%	—

Notes

—: not provided.

Supplementary References

1. Sylvestre JP, Díaz-Marín C, Delgado-Charro MB, Guy RH. Iontophoresis of dexamethasone phosphate: Competition with chloride ions. *J. Control. Release* **131**, 41-46 (2008).

2. Li Y, Yang J, Zheng Y, Ye R, *et al.* Iontophoresis-driven porous microneedle array patch for active transdermal drug delivery. *Acta Biomater.* **121,** 349-358 (2021).

3. Zhao F, Fan S, Ghate D, Romanova S, Bronich TK, Zhao S. A hydrogel ionic circuit based high-intensity iontophoresis device for intraocular macromolecule and nanoparticle delivery. *Adv. Mater.* **34**, 2107315 (2022).

4. Tari K, Khamoushian S, Madrakian T, *et al.* Controlled transdermal iontophoresis of insulin from water-soluble polypyrrole nanoparticles: An in vitro study. *Int. J. Mol. Sci.* **22**, 12479 (2021).

5. Yang Y, et al. Self-powered controllable transdermal drug delivery system. *Adv. Funct. Mater.* **31**, 2104092 (2021).

6. Zhao C, et al. Highly efficient in vivo cancer therapy by an implantable magnet triboelectric nanogenerator. *Adv. Funct. Mater.* **29**, 1808640 (2019).

7. Liu G, et al. Flexible drug release device powered by triboelectric nanogenerator. *Adv. Funct. Mater.* **30**, 1909886 (2020).

8. Ouyang Q, et al. Self-powered, on-demand transdermal drug delivery system driven by triboelectric nanogenerator. *Nano Energy* **62**, 610-619 (2019).

9. Liu Z, et al. Self-powered intracellular drug delivery by a biomechanical energy-driven triboelectric nanogenerator. *Adv. Mater.* **31**, e1807795 (2019).

10. Wu C, et al. Self-powered iontophoretic transdermal drug delivery system driven and regulated by biomechanical motions. *Adv. Funct. Mater.* **30**, 1907378 (2019).

11. Song P, et al. A self-powered implantable drug-delivery system using biokinetic

energy. *Adv. Mater.* **29,** 1605668 (2017).

12. Zhou M, et al. A self-powered "sense-act-treat" system that is based on a biofuel cell and controlled by boolean logic. *Angew. Chem. Int. Ed.* **51**, 2686-2689 (2012).
13. Xiao X, McGourty KD, Magner E. Enzymatic biofuel cells for self-powered, controlled drug release. *J. Am. Chem. Soc.* **142**, 11602-11609 (2020).
14. Ogawa Y, et al. Organic transdermal iontophoresis patch with built-in biofuel cell. *Adv. Healthcare Mater.* **4**, 506-510 (2015).
15. Wang L, et al. A glucose/O2 fuel cell-based self-powered biosensor for probing a drug delivery model with self-diagnosis and self-evaluation. *Chem. Sci.* **9**, 8482-8491 (2018).
16. Hanashi T, Yamazaki T, Tanaka H, Ikebukuro K, Tsugawa W, Sode K. The development of an autonomous self-powered bio-sensing actuator. *Sens. Actuators B Chem.* **196**, 429-433 (2014).
17. Cui Q, et al. A self-powered battery-driven drug delivery device that can function as a micromotor and galvanically actuate localized payload release. *Nano Energy* **66**, 104120 (2019).
18. Ge D, et al. Coating metals on cellulose–polypyrrole composites: A new route to self-powered drug delivery system. *Electrochem. Commun.* **12**, 1367-1370 (2010).
19. Lim C, Hong J, Jung J, Shin Y, Kim DH. Tissue-like skin-device interface for wearable bioelectronics by using ultrasoft, mass-permeable, and low-impedance hydrogels. *Sci. Adv.* **7**, 3716-3723 (2021).
20. Wei G, Dong R, Thamphiwatana S, Li J, Wang J. Artificial micromotors in the mouse's stomach: A step toward in vivo use of synthetic motors. *ACS Nano* **9**, 117-123 (2015).
21. An YH, *et al.* Facilitated transdermal drug delivery using nanocarriers-embedded

electroconductive hydrogel coupled with reverse electrodialysis-driven iontophoresis. *ACS Nano* **14**, 4523-4535 (2020).

22. Gan D*, et al.* Conductive and tough hydrogels based on biopolymer molecular templates for controlling in situ formation of polypyrrole nanorods. *ACS Appl. Mater. Interfaces* **10**, 36218-36228 (2018).

23. Kleber C, Lienkamp K, Ruhe J, Asplund M. Electrochemically controlled drug release from a conducting polymer hydrogel (PDMAAp/PEDOT) for local therapy and bioelectronics. *Adv. Healthcare Mater.* **8**, e1801488 (2019).

24. Heo DN*, et al.* Multifunctional hydrogel coatings on the surface of neural cuff electrode for improving electrode-nerve tissue interfaces. *Acta Biomater.* **39**, 25-33 (2016).

25. Bagheri B*, et al.* Self-gelling electroactive hydrogels based on chitosan-aniline oligomers/agarose for neural tissue engineering with on-demand drug release. *Colloid. Surface B* **184**, 110549 (2019).

26. Qu J, Liang Y, Shi M, Guo B, Gao Y, Yin Z. Biocompatible conductive hydrogels based on dextran and aniline trimer as electro-responsive drug delivery system for localized drug release. *Int. J. Biol. Macromol.* **140**, 255-264 (2019).

27. Wu C, et al. Injectable conductive and angiogenic hydrogels for chronic diabetic wound treatment. *J. Control. Release* **344**, 249-260 (2022).

28. Qu J, Zhao X, Ma PX, Guo B. Injectable antibacterial conductive hydrogels with dual response to an electric field and pH for localized "smart" drug release. *Acta Biomater.* **72**, 55-69 (2018).

29. Servant A*, et al.* Graphene-based electroresponsive scaffolds as polymeric implants for on-demand drug delivery. *Adv. Healthcare Mater.* **3**, 1334-1343 (2014).

30. Spizzirri UG*, et al.* Spherical gelatin/CNTs hybrid microgels as electro-responsive

drug delivery systems. *Int. J. Pharm.* **448**, 115-122 (2013).

31. Park SY, Kang JH, Kim HS, Hwang JY, Shin US. Electrical and thermal stimulus-responsive nanocarbon-based 3D hydrogel sponge for switchable drug delivery. *Nanoscale* **14**, 2367-2382 (2022).

32. Huang W-C, Lee T-J, Hsiao C-S, Chen S-Y, Liu D-M. Characterization and drug release behavior of chip-like amphiphilic chitosan–silica hybrid hydrogel for electrically modulated release of ethosuximide: an in vitro study. *J. Mater. Chem.* **21** (2011).
