# Supplementary material for: An integrated Mg battery-powered iontophoresis patch for efficient and controllable transdermal drug delivery
Source: Nat Commun. 2023 Jan 18;14:297. doi: 10.1038/s41467-023-35990-7 (PMC9849227; doi:10.1038/s41467-023-35990-7)
Supplement: Supplementary file 3 — Reporting Summary [file 41467_2023_35990_MOESM3_ESM.pdf]

nature portfolio

Corresponding author(s): Xiaoteng Jia, Yaping Tian, Caiyun Wang, Danming Chao

Last updated by author(s): Jan 11, 2023

Reporting Summary

Nature Portfolio wishes to improve the reproducibility of the work that we publish. This form provides structure for consistency and transparency in reporting. For further information on Nature Portfolio policies, see our Editorial Policies and the Editorial Policy Checklist.

Statistics

For all statistical analyses, confirm that the following items are present in the figure legend, table legend, main text, or Methods section.

n/a	Confirmed	
☐	☒	The exact sample size (*n*) for each experimental group/condition, given as a discrete number and unit of measurement
☒	☐	A statement on whether measurements were taken from distinct samples or whether the same sample was measured repeatedly
☐	☒	The statistical test(s) used AND whether they are one- or two-sided<br>*Only common tests should be described solely by name; describe more complex techniques in the Methods section.*
☒	☐	A description of all covariates tested
☒	☐	A description of any assumptions or corrections, such as tests of normality and adjustment for multiple comparisons
☐	☒	A full description of the statistical parameters including central tendency (e.g. means) or other basic estimates (e.g. regression coefficient) AND variation (e.g. standard deviation) or associated estimates of uncertainty (e.g. confidence intervals)
☐	☒	For null hypothesis testing, the test statistic (e.g. *F*, *t*, *r*) with confidence intervals, effect sizes, degrees of freedom and *P* value noted<br>*Give P values as exact values whenever suitable.*
☒	☐	For Bayesian analysis, information on the choice of priors and Markov chain Monte Carlo settings
☒	☐	For hierarchical and complex designs, identification of the appropriate level for tests and full reporting of outcomes
☒	☐	Estimates of effect sizes (e.g. Cohen's *d*, Pearson's *r*), indicating how they were calculated

Our web collection on statistics for biologists contains articles on many of the points above.

Software and code

Policy information about availability of computer code

Data collection	OLYMPUS cellSens Standard software (ver. 1.12) were used to acquire images.
Data analysis	Data analysis was performed with GraphPad Prism Software (GraphPad Prism Inc., version 9) and Origin 2017.

For manuscripts utilizing custom algorithms or software that are central to the research but not yet described in published literature, software must be made available to editors and reviewers. We strongly encourage code deposition in a community repository (e.g. GitHub). See the Nature Portfolio guidelines for submitting code & software for further information.

Data

Policy information about availability of data

All manuscripts must include a data availability statement. This statement should provide the following information, where applicable:

- Accession codes, unique identifiers, or web links for publicly available datasets
- A description of any restrictions on data availability
- For clinical datasets or third party data, please ensure that the statement adheres to our policy

The authors confirm that the data of this study are available within the article or its Supplementary information or source data.

Human research participants

Policy information about studies involving human research participants and Sex and Gender in Research.

Reporting on sex and gender	N/A
Population characteristics	N/A
Recruitment	N/A
Ethics oversight	N/A

Note that full information on the approval of the study protocol must also be provided in the manuscript.

Field-specific reporting

Please select the one below that is the best fit for your research. If you are not sure, read the appropriate sections before making your selection.

☒ Life sciences ☐ Behavioural & social sciences ☐ Ecological, evolutionary & environmental sciences

For a reference copy of the document with all sections, see nature.com/documents/nr-reporting-summary-flat.pdf

Life sciences study design

All studies must disclose on these points even when the disclosure is negative.

Sample size	Each finding was confirmed with minimum necessary number such as 3-5 replicates for each experiments. The sample was randomly choose to be determined. These sample sizes are sufficient which have reached statistical significance.
Data exclusions	Data were not excluded from analysis.
Replication	All experiments were repeated 3-5 times independently with similar results.
Randomization	All samples were randomly allocated into experimental groups.
Blinding	Investigators were blinded to group allocation during data collection and analysis.

Reporting for specific materials, systems and methods

We require information from authors about some types of materials, experimental systems and methods used in many studies. Here, indicate whether each material, system or method listed is relevant to your study. If you are not sure if a list item applies to your research, read the appropriate section before selecting a response.

Materials & experimental systems

n/a	Involved in the study	
☒	☐	Antibodies
☐	☒	Eukaryotic cell lines
☒	☐	Palaeontology and archaeology
☐	☒	Animals and other organisms
☒	☐	Clinical data
☒	☐	Dual use research of concern

Methods

n/a	Involved in the study	
☒	☐	ChIP-seq
☒	☐	Flow cytometry
☒	☐	MRI-based neuroimaging

Eukaryotic cell lines

Policy information about cell lines and Sex and Gender in Research

Cell line source(s)	Mouse fibroblast cells (L929 cells) were purchased from Cell Bioscience Inc.
Authentication	L929 cells were not authenticated by the investigators in this paper. L929 cells were purchased from company.
Mycoplasma contamination	L929 cells were not tested for mycoplasma contamination.

Commonly misidentified lines (See ICLAC register)	No commonly misidentified lines were used in the study.

Animals and other research organisms

Policy information about studies involving animals; ARRIVE guidelines recommended for reporting animal research, and Sex and Gender in Research

Laboratory animals	BALB/c mice (5 weeks) and male Spragur-Dawley rats (SD rats, 10 weeks, 300-350 g) were purchased from Liaoning Changsheng Biotechnology Co. Ltd. (Benxi,China). Mice were housed at 23±2 °C, 50% humidity, 20 lux light intensity, and a light/dark alternation time of 12 h.
Wild animals	No wild animals were used in the study
Reporting on sex	Imiquimod-induced psoriasis model in mice is not restricted by the sex of the mice. Therefore, the sex of mice selected for this study was randomized. The skin of hairless male rats was selected for the transdermal delivery experiment.
Field-collected samples	This study did not involve samples collected from field.
Ethics oversight	Institutional Animal Care and Use Committee (IACUC) of Jilin University. The permit number is SY202206009.

Note that full information on the approval of the study protocol must also be provided in the manuscript.
